# Barriers and Challenges to Treatment Alternatives for Early-Stage Cervical Cancer in Lower-Resource Settings

**DOI:** 10.1200/JGO.2016.007369

**Published:** 2017-05-11

**Authors:** Emily S. Wu, Jose Jeronimo, Sarah Feldman

**Affiliations:** **Emily S. Wu**, University of Washington; **Jose Jeronimo**, PATH, Seattle, WA; and **Sarah** **Feldman**, Brigham and Women’s Hospital, Harvard Medical School, Boston, MA.

## Abstract

Cervical cancer is one of the most common cancers among women worldwide, and approximately 85% of new diagnoses occur in less-developed regions of the world. Global efforts in cervical cancer to date have focused on primary and secondary prevention strategies of human papillomavirus vaccination and cervical cancer screening. Cervical cancer screening is effective to reduce the incidence of cervical cancer and can result in diagnosis at earlier stages, but it will take time to realize its full impact. With expansion of screening programs, there is now a greater imperative to increase access to treatment for women who have cervical cancer, particularly in earlier stages of disease, when it is still curable. Resources for multimodality treatment can be limited—or even absent—in many less-developed regions of the world and may be associated with geographic, social, and financial barriers for the patient. However, there is evidence that, in many cases, less-invasive and less–resource-intensive treatment options are still effective. To this end, the National Comprehensive Cancer Network and American Society of Clinical Oncology have published guideline adaptations for specific resource constraints, and research about more conservative approaches to the treatment of cervical cancer continues. This review focuses on potential barriers and challenges to provision of safe and effective treatment of early-stage cervical cancer in lower-resource settings, and it suggests future directions for expansion of access to cervical cancer treatment around the world.

## INTRODUCTION

Cervical cancer is the fourth most common cause of cancer in women worldwide and the most common cancer in women in eastern and middle Africa. Approximately 85% of the 528,000 new diagnoses of cervical cancer and 87% of the 266,000 deaths occur in less-developed regions of the world.^1^ Because of improvements in maternal health, deaths as a result of cervical cancer now outnumber those that are results of maternal mortality in most countries in Asia and Latin America and in some countries in Africa.^2^ Even with increasing availability of cervical cancer screening and vaccination around the world, prevalent occurrences will continue to be identified and to warrant treatment. In screened populations, greater than half of detected cancers diagnosed can be stage I or II, when less-radical treatment strategies are still an option.^3,4^ To address wide variations in the availability of resource-intense and highly technical interventions, such as radical surgery, chemotherapy, and radiation therapy, the National Comprehensive Cancer Network (NCCN) and American Society of Clinical Oncology (ASCO) have published resource-stratified guidelines to delineate appropriate care options for women with cervical cancer. The objective of this review is to discuss barriers and challenges to treatment alternatives for early-stage cervical cancer—stages IA1 to IIA1—in lower-resource settings. Because many lower-resource settings are in Africa, this article largely focuses on the situation there; however, many of the principles and issues we raise also are applicable to many parts of Asia and Latin America.

## CERVICAL CANCER PREVENTION

Cervical cancer can be prevented with human papillomavirus (HPV) vaccination and cervical cancer screening. Primary prevention with the HPV vaccine is expected to result in a significant decrease in the incidence of cervical cancer worldwide. Epidemiologic modeling has estimated that vaccine coverage of 90% could result in a decrease of up to 83% in incident cervical cancer occurrences worldwide.^5^ The HPV vaccine became available in 2006 and is available in approximately one third of low- and middle-income countries (LMICs), but many programs in low-income countries reach less than 10% of the target population.^6^ Although the vaccine is promising, differences in cervical cancer incidence that result from HPV vaccination will take decades to be realized.

In the shorter term, secondary prevention with cervical cancer screening has been shown to prevent malignancy when precancer is detected and treated. Furthermore, prevalent occurrences of invasive cancer are detected at earlier stages. In India, a cluster-randomized trial of 30,577 women compared a single round of visual inspection with acetic acid (VIA)–based screening versus no screening and found that, in the screened arm, 35% of cervical cancer occurrences were stage I, and 18% were stage II, compared with 0% and 6%, respectively, in the control arm.^3^ Cytology is the most commonly used screening method in developed countries, but see-and-treat VIA is used frequently in lower-resource settings.^7^ Greater than 50 low-income countries have introduced cervical cancer screening with VIA.^8^

Although the availability of cervical cancer screening programs has been increasing, coverage is still low. Most screening programs in low-income countries and the WHO African region are estimated to reach less than 10% of the population.^6^ Therefore, as screening programs scale, they likely will continue to identify a large number of prevalent invasive cancer occurrences that require treatment.

## RESOURCE-STRATIFIED GUIDELINES FOR THE TREATMENT OF CERVICAL CANCER

Cervical cancer is clinically, rather than surgically, staged via the International Federation of Gynecology and Obstetrics (FIGO) system^9^ (Table 1). Treatment of early-stage invasive cervical cancer historically has included surgery, such as cold knife conization, simple hysterectomy, or radical hysterectomy with pelvic lymph node dissection.^10,11^ More advanced disease generally is treated with chemoradiation.^11,12^ However, radical surgery, chemotherapy, and/or radiation are not available in many parts of the world. They are expensive modalities and require highly trained personnel and quality assurance, which may not be realistic or feasible in some locations. For example, surgical treatment of locally advanced disease is typically a radical hysterectomy, which, in contrast to a simple hysterectomy, involves removal of parametrial tissue and an additional vaginal margin. The surgery is technically more difficult to perform, requires more specialized training, and carries a higher risk of operative (eg, bleeding, infection, and injury) and long-term (eg, bladder dysfunction and fistula) complications. As a result, there is increasing research on more conservative approaches to the treatment of cervical cancer.^13^ In lower-resource settings, options such as cold-knife conization, simple hysterectomy, or neoadjuvant chemotherapy followed by simple hysterectomy may provide more realistic options for cure.

**Table 1:**
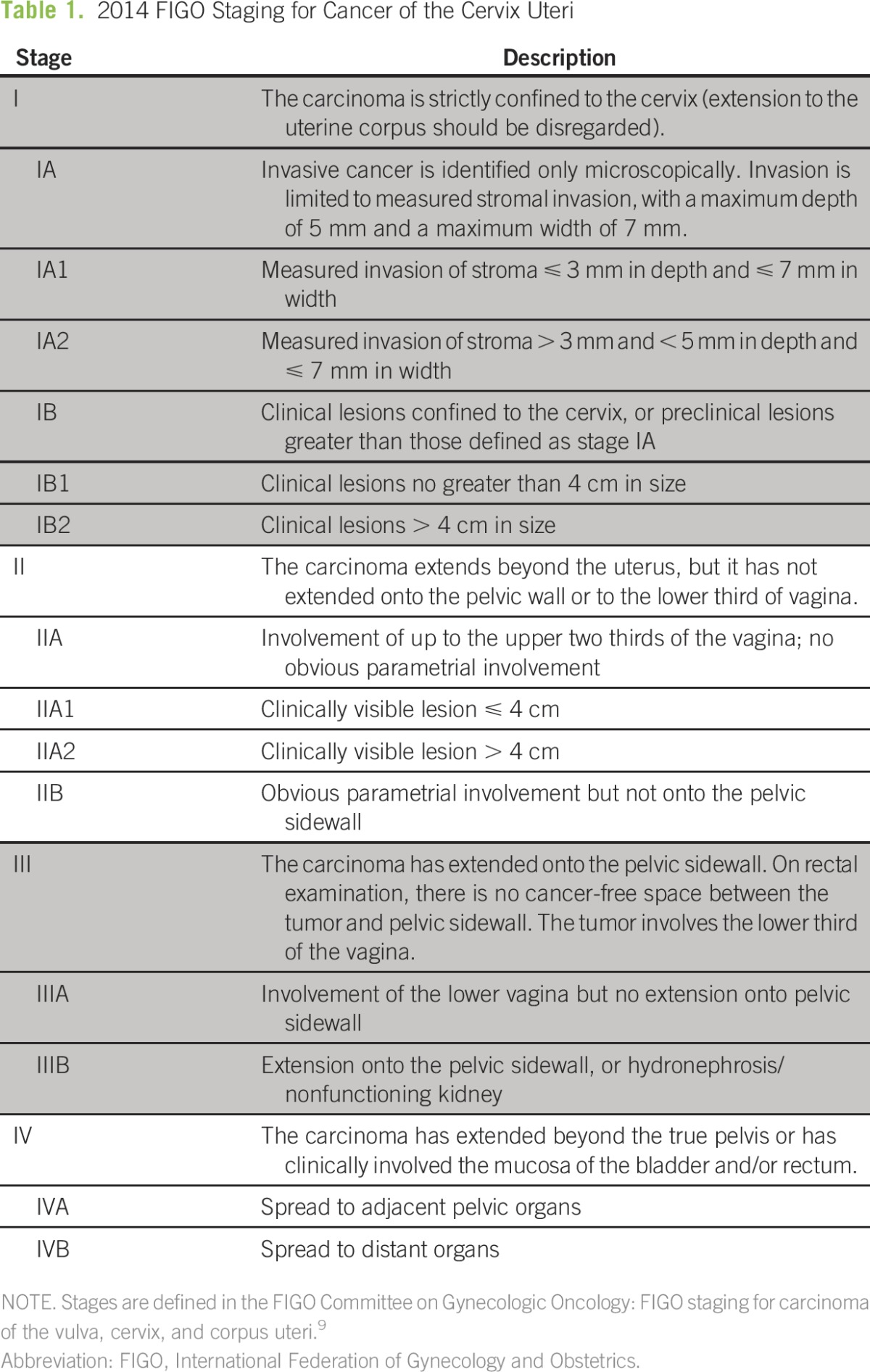
2014 FIGO Staging for Cancer of the Cervix Uteri

The NCCN guidelines provide multiple options to treat each cervical cancer stage (Table 2), including options for fertility-sparing treatment in early stages.^11^ Treatment of stage IA disease generally is less invasive and can be adapted to lower-resource settings. However, patients in lower-resource settings are more likely to present at stages for which the recommended treatment modality is not readily available.^14-16^ Therefore, both the NCCN (in the NCCN Framework) and ASCO created resource-stratified guidelines for women with invasive cervical cancer (Table 2).^17,18^ Both guidelines outline recommendations for each of four resource levels: basic, limited, enhanced, and maximal. For example, although NCCN guidelines recommend a radical hysterectomy with pelvic lymph node dissection or chemoradiation with brachytherapy for stage IB1 disease, the NCCN Framework guidelines recommend a simple hysterectomy or radical hysterectomy with pelvic lymph node dissection for basic-level settings; ASCO recommends a simple hysterectomy with or without neoadjuvant chemotherapy at that same level.

**Table 2:**
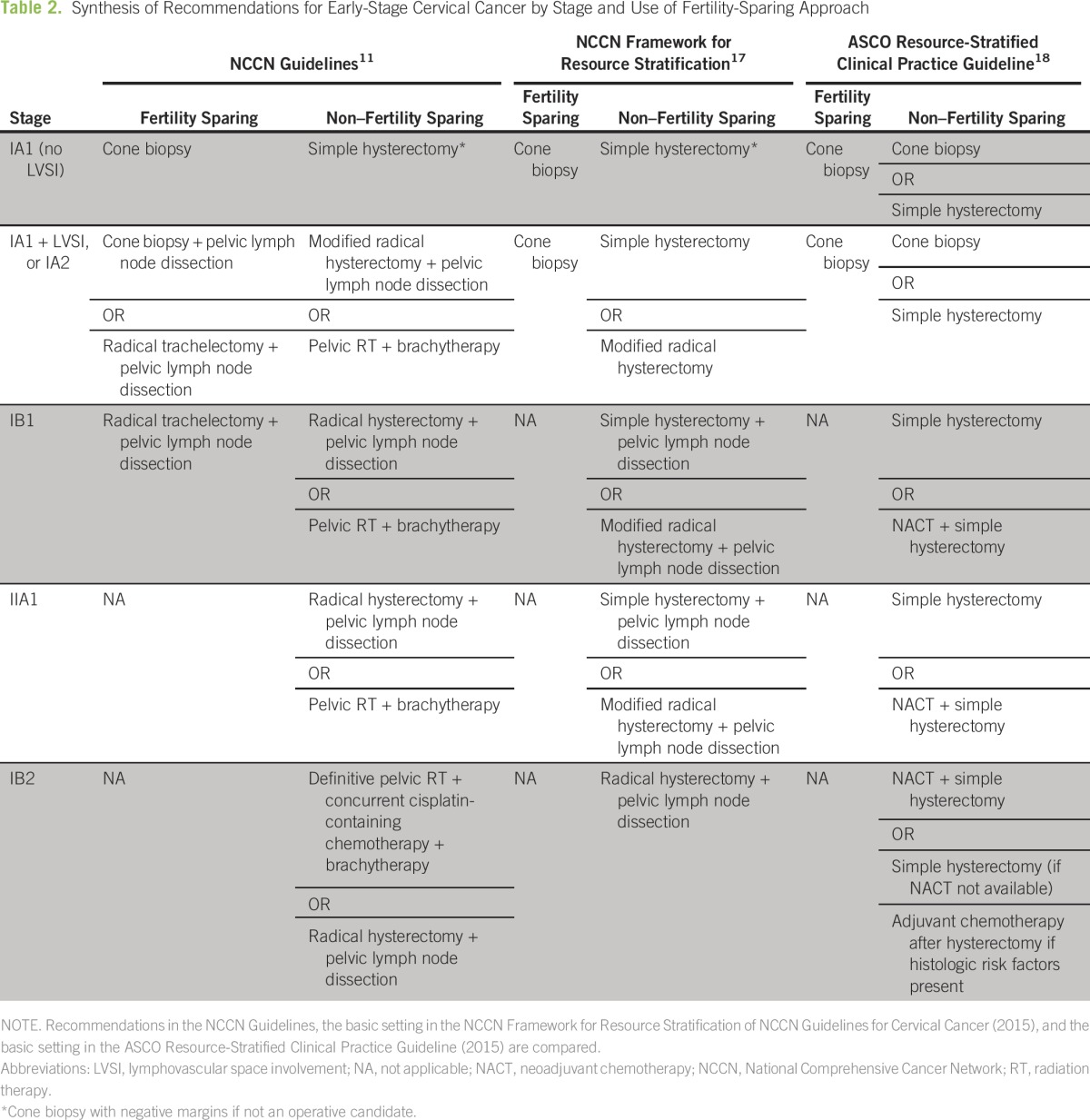
Synthesis of Recommendations for Early-Stage Cervical Cancer by Stage and Use of Fertility-Sparing Approach

## RESOURCES CURRENTLY AVAILABLE IN LOWER-RESOURCE SETTINGS

### Surgery

Adequate training and access to appropriate providers have been ongoing limitations in global health. Compared with high-income countries, which have 28.7 physicians per 10,000 people, low-income countries have only 2.5 physicians per 10,000 people.^19^ Low-income countries have an estimated 0.7 surgical providers per 100,000 people compared with 56.9 in high-income countries.^20^

There are fewer statistics on capacity for gynecologic surgery. A review of the loop electrosurgical excision procedure (LEEP) in lower-resource countries found that this procedure usually is performed by physicians.^21^ However, it is feasible to train nonphysicians to perform the procedure safely, and such training may represent a task-shifting opportunity. A program in Kisumu, Kenya, has successfully trained and certified clinical officers (the equivalent of a physician assistant) to perform LEEPs.^22^

Data about capacity to perform simple hysterectomies, radical hysterectomies, and lymph node dissections in lower-resource settings are scarce. Many providers who currently perform hysterectomies in lower-resource settings are primary care physicians who have undergone only 1 year of surgical training. Radical hysterectomy is not available in many lower-resource settings or may be available only in large central hospitals.^23,24^ Many professional societies and nongovernmental organizations have initiated independent volunteer-based efforts in different countries to expand this capacity through didactics and hands-on mentorship in Africa, Central America, and Asia.^25-27^ Although efforts by voluntary organizations to improve training of local providers are laudable, formal teaching programs within each country must be developed and supported locally, either by providing in-country training or by sending qualified candidates abroad.

Even when surgical services exist, they often are concentrated in referral hospitals in urban areas, which may result in barriers related to cost,^28^ transport, long wait times, poor referral networks, and inability to pay. Patients also may be hesitant to undergo surgery because of fear of the procedure, fear of anesthesia, and rumors of poor outcomes.^29^ Even if trained surgical personnel exist, surgical services may be intermittently available because of supply and medicine stockouts, power shortages, inconsistent water supply, and poor infrastructure.^30^

### Radiation Therapy

There are inadequate personnel and equipment to meet the demand for radiation therapy in lower-resource settings.^31^ The worldwide standard for the number of radiation megavoltage machines is one per 700,000 to 800,000 people or per 350 to 400 patients with cancer.^32^ In a recent survey of radiotherapy capacity, the average number of teletherapy machines per million people was 0.21 for low-income countries compared with 8.6 for high-income countries.^33^ Of the 52 African countries in this survey, only 23 offered external-beam radiotherapy, and only 20 offered brachytherapy. Combined, these countries housed 88 cobalt-60 units and 189 linear accelerators; however, 60% of these machines were concentrated in South Africa (n = 92 machines) and Egypt (n = 76 machines). Most radiotherapy centers only provided basic services, such as palliation and simple curative treatments, on the basis of two-dimensional imaging and treatment planning.^33^ A separate survey of 17 countries in the Asia and Pacific region reported 0.09 to 7.39 megavoltage machines per million people.^34^ Only four countries—Australia, Japan, New Zealand, and Singapore—exceeded two machines per million people. In this survey, many departments reported treatment of patients without simulators or treatment-planning systems. Furthermore, radiation oncologists often had additional duties, such as medical oncology, medical physics, or diagnostic radiology duties.^34^ In Latin America, the most recent survey is from 2004 and identified 470 radiotherapy centers in the region, which had 710 machines across 19 countries. The distribution of centers ranged from none to 151 per country surveyed.^31^

Efforts to increase radiotherapy capacity in lower-resource settings have been increasing. The International Atomic Energy Agency has been involved in projects to establish and improve radiotherapy in countries around the world.^35^ The International Education Subcommittee of the American Society of Radiation Oncology has collaborated with sister societies around the world to foster education.^36^

Because of the complexity in the establishment of safe infrastructure and the training of specialized teams that include radiation oncologists, physicists, therapists, and nurses, radiotherapy services likely will exist only in urban areas. In addition to the difficulty of obtaining transport, patients may face barriers related to being away from home for extended periods of time for their course of radiotherapy. Radiotherapy also is expensive—the capital costs of a linear accelerator can be greater than 1 million US dollars (USD), and those of a cobalt machine can be up to $480,000 USD. The median annual cost of quality assurance and maintenance of a linear accelerator has been estimated as $41,000 USD; that of a cobalt machine, $6,000 USD. These costs do not include power, personnel, and building costs.^37^

### Chemotherapy

In cervical cancer, chemotherapy can be used as neoadjuvant therapy, as adjuvant therapy, or as a sensitizing agent for radiation therapy. A recent study about national essential medicine lists from LMICs found that most lists contained multiple oncology medicines.^38^ However, it is unclear how this has translated into availability. Access likely is limited: the African Palliative Care Association has estimated that only 5% of patients with cancer in Africa receive chemotherapy.^39^ In Southeast Asia, an estimated 15% of patients from LMICs in the region have access to essential oncology medications.^40^

In addition to barriers, such as patient fear of chemotherapy, distance from infusion centers, and poor referral networks, patients in LMICs likely face difficulty with schedules and payments for chemotherapy. A study in Cameroon found that 24% of patients experienced a delay in receipt of chemotherapy because of finances, and 38% were unable to schedule or keep a chemotherapy appointment in a timely manner. A total of 40% of patients spent greater than $200 USD on the most recent round of chemotherapy.^41^ This is a significant fraction of the gross national income per capita of $1,026 USD to $4,035 USD that defines the World Bank classification of lower and middle income.^42^ The Clinton Health Access Initiative and American Cancer Society recognized cost as a major obstacle to timely and high-quality cancer care worldwide and established a partnership in 2015 to improve capacity for cancer treatment. With an initial focus on breast and cervical cancer, this initiative seeks to strengthen capacity at tertiary hospitals and optimize the market for cancer drugs (eg, chemotherapy) to expand access to quality and affordable treatment.^43^

### Pathology

Adequate pathology services are crucial to provision of oncology care, both to confirm malignancy and to determine the best treatment. In addition to human resource and training requirements for clinical pathologists and laboratory technicians, sufficient infrastructure, equipment, maintenance contracts, and reagents to properly transport, fix, and process tissue for histologic analysis are vital. In sub-Saharan Africa, there are 84,133 to 9,264,500 people per pathologist, which is a much higher ratio than the 15,000 to 20,000 people per pathologist in the United Kingdom or the United States. Of 30 sub-Saharan African countries that reported data, immunohistochemistry was available in 16 and molecular diagnostics, in two.^44^ The scarcity of pathology services may be due in part to the perception that pathology services are restricted to services provided by laboratory technicians rather than by medically trained clinicians.^45^

In 2014, the African Strategies for Advancing Pathology group was created with the goal to develop “a robust framework for efforts to increase and improve pathology services within sub-Saharan Africa.”^44(p24)^ Other international partners also are investing in improvements to pathology capacity in lower-resource settings.^46^

### Research Challenges

Many less-invasive treatment options for cervical cancer, such as those recommended in the NCCN Framework and ASCO resource-stratified guidelines, are supported by small observational studies. Because of a lack of high-level evidence for treatments feasible in more resource-limited settings, many recommendations are based on expert consensus.^17,18^ Although most of the disease burden is in more resource-limited areas, cervical cancer research is conducted disproportionately in resource-rich settings. Therefore, a major challenge to extension of access to cervical cancer treatment—particularly alternatives suitable for resource-limited settings—is a strong evidence base. Prospective studies with adequate sample size and power to evaluate the efficacy and feasibility of proposed treatment alternatives for early-stage cervical cancer are necessary. In the interim, there is an urgency to start implementation of innovative treatment strategies, because women with potentially curable cancer continue to die without any treatment.

### Logistic and Social Challenges

It may take years to develop the human resources, implement the systems of care, and procure the supplies needed to provide the current resource-intense standard of care for cervical cancer. Even in settings where resources may be available, access continues to be a challenge, and poor access can lead to delays in care and poorer outcomes.^47^ For example, a study in New Delhi reported a median of 41 days from registration to radiation therapy initiation; 25% of patients did not complete therapy.^48^ Many African countries, including Cameroon, Rwanda, and South Africa, have reported an interval of up to 7 months between request for care as a result of symptoms and treatment of cancer.^41,49,50^ Although more than half of the population in less-developed countries reside in rural areas,^51^ oncology services tend to exist in urban areas. This means that many patients must travel long distances for treatment and face competing pressures among cost of travel and treatment, family obligations, and work responsibilities.

At the tertiary-care level, multidisciplinary management will be essential to ensure continuity of care, use of appropriate treatment protocols, and management of adverse effects and complications from treatment. When patients present for care, systems should be put in place to ensure that patients receive a timely histologic diagnosis, are not lost to follow-up because of confusion about multiple treatment modalities, receive referrals for adjuvant therapy in a timely manner, and benefit from counseling to understand the nature of the illness and the rationale for treatment.

### Progress to Date

Although the literature is dominated by reports of limitations in oncology services and barriers to implementation, it is important to acknowledge the progress that has been made. In 2012, the African Organization for Research and Training in Cancer launched the African Cancer Network Project. As part of the project, a partial list of cancer treatment institutions in Africa was compiled and included 102 centers.^52^ Some of these centers may already be, or could become, research and training hubs to help serve their regions, such as Uganda, Bangladesh, and Rwanda.^53^

At a policy level, 79% of low-income countries and 84% of LMICs have a cancer policy, strategy, or plan. Although only 45% and 58%, respectively, have an operational policy with funding, the policy efforts represent aspirations to improve oncology services.^54^ Civil society is taking an active role through community awareness, early detection campaigns, and advocacy. Many efforts are survivor led and focus on breast and cervical cancer.^55^

## OPTIONS TO TREAT EARLY-STAGE CERVICAL CANCER IN LOWER-RESOURCE SETTINGS

Early-stage cervical cancer spreads by local extension to the endocervix, uterine corpus, parametrium, and vagina. It also can spread via lymphatic channels to the pelvic lymph nodes, which confers a worse prognosis.^56^ This risk serves as the rationale in higher-resource settings for evaluation or treatment of the parametria and pelvic lymph nodes with radical hysterectomy and pelvic lymph node dissection in stages as early as IA1 with lymphovascular space invasion (LVSI). In a classic study of patients with stage I disease treated with radical hysterectomy and pelvic lymphadenectomy, tumor size, depth of invasion, and LVSI were independent prognostic factors for survival.^57^ The 3-year disease-free survival (DFS) rate was 85.6% with negative nodes and was 74.4% with positive nodes. DFS in patients with positive parametria was 69.6% and was 84.9% in patients with negative parametria. DFS was 69.1% in patients with positive margins and was 84.3% in patients with negative margins.^57^ Ideally, the goals of surgical therapy are to excise tissue at risk for disease, achieve negative margins, and determine the need for additional therapy. In well-resourced settings, the standard of care involves removal of any at-risk areas, including the parametria and pelvic lymph nodes; yet, many patients with early-stage disease do not have involvement of parametrium or nodes and could theoretically be cured with less-radical surgery. An increasing number of studies have provided evidence for less-radical surgery in patients with stage IA2 and IB1 disease,^58-60^ in whom the risk of parametrial involvement is approximately 2% and 6% to 10%, respectively and the risk of pelvic lymph node metastases is less than 15% (Table 3).^61-64^

**Table 3:**
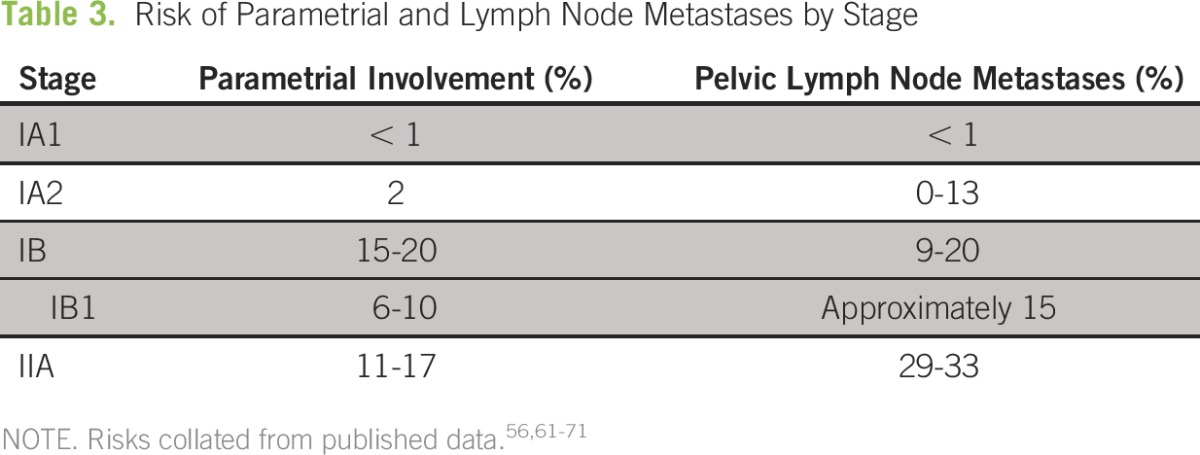
Risk of Parametrial and Lymph Node Metastases by Stage

### LEEP

In the absence of other services, a LEEP procedure could be used in stages IA1, IA2, and IB1 disease smaller than 2 centimeters. Studies of conservative treatment of stages IA1 to IB1 disease ranged in eligibility criteria and use of adjuvant treatment and may have included imaging modalities (eg, magnetic resonance imaging) that are not readily available in lower-resource settings. However, outcomes from these studies suggest their feasibility. An analysis of 1,409 patients with stage IA1 cervical cancer reported a 5-year survival of 98% versus 99% (hazard ratio, 0.65; 95% CI, 0.23 to 1.47) in women who underwent a cold knife conization versus those who underwent a hysterectomy.^72^ In IA2 disease, one study reported a 98% survival rate in 66 women who underwent a cold knife conization after a median follow-up time of 19 years. Twenty-eight of these patients underwent lymphadenectomy, and no positive lymph nodes were identified.^73^ Maneo et al^74^ used cold knife conization and pelvic lymph node dissection to treat a selected group of patients with stage IB1 tumors smaller than 2 centimeters without evidence of enlarged lymph nodes or uterine involvement. After a median of 66 months, one recurrence was noted 34 months after initial treatment. No positive lymph nodes were found.^74^

Per the NCCN guidelines, a LEEP rather than a cold knife conization is acceptable if specimen integrity with adequate margins can be obtained. The procedure is relatively simple, can be done in the clinic, does not require a physician, and has established feasibility and safety in LMICs.^22,75,76^ To study this question more, two prospective studies through MD Anderson Cancer Center and the Gynecologic Oncology Group are underway to evaluate simple hysterectomy or cone biopsy with pelvic lymphadenectomy in early-stage cervical cancer.^60,77^

### Neoadjuvant Chemotherapy

In larger tumors for which surgery may not be sufficient or worthwhile without other treatment modalities, neoadjuvant chemotherapy to reduce the tumor burden to enable surgical excision can be considered. This approach is recommended throughout the ASCO resource-stratified guidelines.

One application for this approach could be neoadjuvant chemotherapy followed by a cold knife conization, although the evidence is limited to small studies of highly selected women, some of whom ultimately underwent more radical surgery.^78,79^ Another application of neoadjuvant chemotherapy could be to downstage the tumor in more advanced disease before a less radical surgery, such as a simple hysterectomy. Few studies validate this approach, and they typically are limited to the fertility-sparing setting—for example, neoadjuvant chemotherapy followed by laparoscopic lymphadenectomy and vaginal simple trachelectomy.^80^

It is important to emphasize that neoadjuvant chemotherapy currently is not standard treatment and that the workup involved in determination of eligibility, in itself, can involve resources that are difficult to access in lower-resource settings. However, if resource-appropriate selection criteria can be established, the number of patients who may be able to obtain treatment would increase, particularly if more aggressive treatments are not easily accessible. Ultimately, the choice of surgical procedure should be tailored to the setting of each patient and should be predicated on what surgical care is safely available for the cancer stage of the patient and whether appropriate supplies, support systems, and facilities are available.

## FUTURE DIRECTIONS

**Fig 1:**
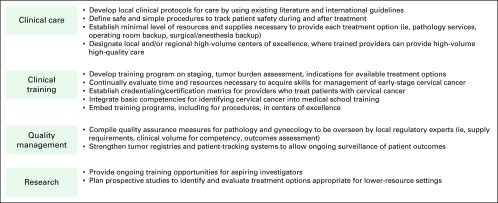
Future directions to increase access to cervical cancer treatment in lower-resource settings include attention to clinical care, clinical training, quality management, and research.

The current global effort to prevent and detect cervical cancer continues to scale. Screening programs ultimately will decrease cervical cancer incidence after the initiation of screening and also will downshift the stage distribution of occurrences if treatment becomes more widely available.^3^ As awareness and advocacy about cervical cancer increase, so does the imperative to provide access to treatment. In many situations, the default often is to do nothing, which means certain death. Some resources are available, but they are not yet available to the same degree as in high-income economies. Doing nothing should not be an option; therefore, researchers and policy makers should focus their activities on how best to balance the use of existing resources with the expected impact on quantity and quality of life (Fig 1). Clinicians should use existing international guidelines, such as the NCCN Framework and ASCO resource-stratified guidelines, to provide the maximally feasible treatment option. The medical and policy communities should measure outcomes to ensure that good care is being provided, identify areas for improvement, and prioritize research activities. Future research priorities in LMICs can focus on identification of more resource-appropriate alternatives to the current treatment paradigms and on strategies to better operationalize access to prevention and treatment.

In conclusion, although cervical cancer screening and prevention programs have been growing, cervical cancer still is prevalent, and treatment has not become widespread. Rather, women often are referred to palliative care and are condemned to death. Although the traditional standard of care for early-stage cervical cancer has been radical surgery or chemoradiation, there are data to suggest less-invasive, and therefore potentially more accessible, treatments. The NCCN Framework and ASCO have published resource-stratified guidelines with alternative treatment recommendations that can guide countries in applications of their available resources to cervical cancer treatment. For each patient, these guidelines should be tailored to the extent of the disease, the surgical procedures that can be safely performed, and other available treatment modalities. Although many gaps in oncology resources and barriers to treatment exist, there is increased political will and international attention to improving access to safe and effective treatment of cervical cancer.
